# Clinical and Genetic Heterogeneity in a Cohort of Chinese Children With Dopa-Responsive Dystonia

**DOI:** 10.3389/fped.2020.00083

**Published:** 2020-02-28

**Authors:** Yan Chen, Xinhua Bao, Yongxin Wen, Jiaping Wang, Qingping Zhang, Jiayou Yan

**Affiliations:** Department of Pediatric, Peking University First Hospital, Beijing, China

**Keywords:** dopa-responsive dystonia, L-dopa, genetic test, clinical and genetic heterogeneity, prognosis of dopa-responsive dystonia

## Abstract

**Background:** The aim of this study was to investigate the genetic and clinical features of dopa-responsive dystonia (DRD) in China.

**Method:** Characteristics of gene mutations and clinical manifestations of 31 patients diagnosed with DRD were analyzed retrospectively.

**Result:** From January 2000 to January 2019, 31 patients were diagnosed with DRD. Twenty (64.5%) were male, and 11 (35.5%) were female. Ten patients (32.3%) had classic DRD, 19 (61.3%) had DRD-plus, and 2 (6.4%) patients had mutations in the dopamine synthetic pathway (*PTS* gene mutation) without a typical phenotype (not DRD or DRD-plus). Twenty-eight (90.3%) patients underwent genetic testing. Homozygous or compound heterozygous *TH* gene mutations were found in 22 patients. *GCH1* and *PTS* gene mutations were found in 2 patients. Heterozygous *TH* mutation and genetic testing were negative in 1 patient. They took different doses of L-dopa, ranging from 0.4 to 8.7 mg/kg/d. Patients with classic DRD responded well. In patients with DRD-plus, 94.7% (18/19) responded well with residual symptoms. One patient (5.3%) did not show any improvement.

**Conclusion:** DRD can be divided into classic DRD and DRD-plus. In this cohort, the most common pathogenic gene was *TH*. Fever was the important inducing factor of the disease. L-dopa has sustained and stable effects on patients with classic DRD. In patients with DRD-plus, treatment with L-dopa could ameliorate most of the symptoms.

## Introduction

Dopa-responsive dystonia (DRD) is clinically defined as a kind of hereditary progressive dystonia with marked diurnal fluctuation in tradition. However, with the recognition of the disease, many atypical manifestations have been reported. These issues caused confusion as to what DRD is. For this reason, Jeon et al. suggested that DRD should be genetically defined as a syndrome of selective nigrostriatal dopamine deficiency caused by genetic defects in the dopamine synthetic pathway without nigral cell loss (1). The estimated prevalence of the disease is 0.5–1 per million (2, 3). DRD was first described by Segawa et al. (4). Thereafter, classic DRD has been well-recognized. In recent years, patients with atypical manifestations have been reported. The onset of the disease could be as early as the neonatal period. Some patients had psychomotor retardation, convulsion, and parkinsonism, which may be accompanied by vegetative and fluctuating extrapyramidal symptoms (5–7). For this reason, Jeon et al. proposed the term “DRD-plus” to cover patients with features that were not seen in classic DRD in 1988 (1, 8). Classic DRD is described as childhood- or adolescent-onset dystonia associated with diurnal fluctuation, parkinsonism, and a good response to a small dosage of L-dopa (9). However, no relationship has been found between genotype and phenotype. Genes involved in DRD include *GCH1, TH, PTS, SPR, QDPR*, and *PCBD*. Tyrosine hydroxylase (TH EC 1.14.16.2; gene symbol *TH* OMIM 191290) makes dopamine from tyrosine. Tetrahydrobiopterin (BH4) is a cofactor of tyrosine hydroxylase. The enzymes involved in the *de novo* biosynthesis of BH4 include GTP cyclohydrolase 1 (GCH1 EC 3.5.4.16), encoded by the *GCH1* gene (OMIM 600225); pyruvoyl-tetrahydropterin synthase (PTPS EC 4.6.1.10), encoded by the *PTS* gene (OMIM 612719); and sepiapterin reductase (SR EC 1.1.1.153), encoded by the *SPR* gene (OMIM 182125). In addition to the enzymes related to the biosynthesis of BH4, there are two enzymes related to the regeneration of BH4, pterin-4a-carbinolamine dehydratase (PCD EC 4.2.1.96), encoded by the gene *PCBD* (OMIM 126090), and dihydropterin reductase (DHPR EC 1.6.99.7), encoded by the gene *QDPR* (OMIM 612676). Thus, in theory, the deficiency of any of the above enzymes related to the biosynthesis and recycling of BH4 could be the cause of DRD. Furthermore, BH4 is also an essential cofactor for the activity of other enzymes, such as nitric oxide synthases, phenylalanine and tryptophane hydroxylases (10). These enzymes have many functions, which may explain the clinical heterogeneity.

However, very few reports have published the frequency of gene mutations and the relationship between the genotype and phenotype of DRD. In this study, a cohort of 31 Chinese patients diagnosed with DRD was investigated clinically or genetically. Their clinical characteristics and related mutated genes are reported here.

## Patients And Methods

### Patients

#### Inclusion Criteria

Patients with dystonia who visited the Department of Pediatrics, Peking University First Hospital between January 2000 and 2019 were recruited. A low dose of L-dopa was administered. We used Burke-Fahn-Marsden (BFM) to measure patients' dystonia and its variation after L-dopa. Patients who met either clinical criteria or genetic criteria were diagnosed with DRD. Clinical criteria refers to patients whose dystonia improved by at least 50% after the treatment. Genetic criteria refers to patients whose genetic analysis showed mutation of the *GCH1, PTS, SPR, TH, PCBD*, or *QDPR* genes. All patients were divided into classic DRD or DRD-plus clinically. Classic DRD referred to the patients who had isolated dystonia without other neurological manifestations and a sustained response to a low dose of L-dopa. DRD-plus referred to the patients with the following phenotypes: (1) earlier onset than classic DRD, such as neonatal onset; (2) more severe motor phenotypes, such as poor sucking, swallowing difficulties, and severe hypotonia; and (3) non-motor features (extra-nigrostriatal dopaminergic dysfunctions), such as convulsions (generalized convulsion or myoclonic seizures), psychomotor retardation, mental retardation, drowsiness, irritability, recurrent hyperthermia without infections, and ptosis (9).

#### Exclusion Criteria

All patients were re-evaluated regardless of their previous diagnosis. Patients who had neither clinical criteria nor genetic criteria were excluded from our cohort.

### Clinical Data

The clinical data were carefully collected, including family history, gender, age of onset, age of diagnosis, clinical manifestations of dystonia, and additional features at the time of onset, diurnal fluctuation, neurological signs, treatment and prognosis. The end of follow-up was June 2019.

#### Genetic Analysis

Genomic DNA was extracted using standard methods from the peripheral blood of the patients and their parents. Polymerase chain reaction (PCR) analysis of *TH* and *GCH1* was performed before 2016. After 2016, patients were analyzed by a targeted next-generation sequencing (NGS) panel containing 20 genes, including *ANO3, ATP1A3, CACNAA1B, GCH1, GNAL, HPCA, PCBD1, PNKD, PRKRA, PRRT2, PTS, QDPR, SGCE, SLC2A1, SPR, TAF1, TH, THAP1, TOR1A*, and *TUBB4A*, or by whole-exome sequencing. MLPA was performed in case 23 in 2019 because the patient had one point mutation that could not explain his disease. Library preparation was carried out following a standard Ion AmpliSeq library preparation protocol (pub. no. MAN0006735). The enriched libraries were sequenced on the Illumina HiSeq 2500 platform (CA, USA) to generate 100 bp paired-end reads. Raw reads were aligned to UCSC hg19 with BWA software. Aligned reads were processed with SAMtools and Picard following the best practice guidelines of the Genome Analysis Toolkit (GATK). Single-nucleotide variants (SNVs) and small insertion-deletions (indels) were detected with the GATK Haplotype Caller.

Multiplex ligation-dependent probe amplification (MLPA) was performed to evaluate large deletions and duplications using the SALSA MLPA Probemix P099 GCH-1-TH-SGCE. The obtained *TH* and *GCH1* gene products were separated and analyzed using the ABI Prism 3100 Genetic Analyser and GeneScan software according to the manufacturer's instructions.

Variants were annotated with ANNOVAR (http://annovar. openbioinformatics.org/en/latest/). Common sites with a population allele frequency above 5% according to the dbSNP 138, 1000 Genomes Project, ESP6500 or ExAC databases were excluded. Variant pathogenicity was interpreted according to the ACMG Standards and Guidelines of 2015. The pathogenic variants were validated by Sanger sequencing.

## Results

**Figure 1:**
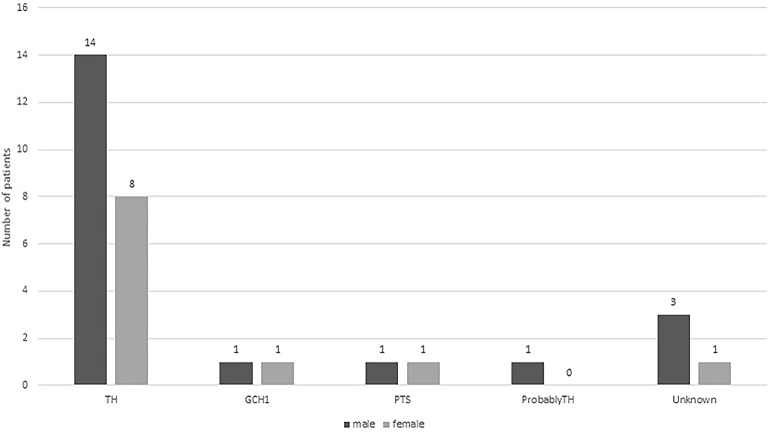
Distributions of boys (dark gray bar) and girls (light gray bars) with different gene mutation.

In total, thirty-one patients were diagnosed with DRD. Among 31 patients, 10 (32.3%) were diagnosed with classic DRD, 19 (61.3%) with DRD-plus, and 2 (6.4%) with 6-pyruvoyl-tetrahydropterin synthase deficiency but without a typical phenotype. Twenty (64.5%) were male, and 11 (35.5%) were female (Figure 1). Eleven of them had a family history of DRD, 10 of them were siblings, and 1 was a daughter and father. Twenty-nine had brain MRI, one (case 11) showed delayed myelination, one showed delayed white matter development, and others were normal. Twenty-eight (90.3%) underwent genetic testing. Twenty-two patients, six of whom were diagnosed with classic DRD and 16 with DRD-plus, had homozygous or compound heterozygous mutations in the *TH* gene. A heterozygous mutation in the *TH* gene was detected in one patient with DRD-plus. Two patients had *GCH-1* mutations and were diagnosed with classic DRD. Two patients had *PTS* gene mutations, and they did not have the clinical features of DRD. Genes related to dopa-responsive dystonia were not detected in one patient. Three patients did not have a genetic test. All patients received different doses of L-dopa, and most responded well, except for 1 patient with DRD-plus caused by *TH* mutation and two patients with 6-pyruvoyl-tetrahydropterin synthase deficiency caused by *PTS* gene mutation.

Unfortunately, cerebrospinal fluid (CSF) neurotransmitters are also very important for the diagnosis of DRD, but we failed to perform these tests. The possible reasons are as follows: (1) many patients came to our hospital many years ago, and although they had lumbar puncture, it was difficult to analyse CSF neurotransmitters at that time; (2) most parents think lumbar puncture injury is very large, and they refused this examination; and (3) compared with lumbar puncture to obtain CSF, parents think genetic testing is more valuable. Once genetic testing has told them the disease of their children, they do not want to have any other tests.

### Patients With Mutations of the TH Gene

**Figure 2:**
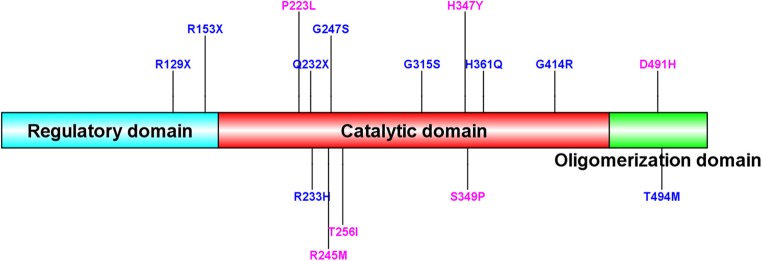
Sequence alignment of human TH (isoform 1) and mutation site of our patients. New TH mutations were drawed in pink color. The protein consists of four subunits. Each subunit is made up of a regulatory ACT domain with an unstructured N-terminal tail of different length, a catalytic domain and an oligomerization domain.

Homozygous or compound heterozygous mutations in the *TH* gene were found in 22 (78.6%) patients, which is consistent with autosomal recessive inheritance. There were 20 different mutations, including 11 missense mutations, four non-sense mutations, three intron codon site mutations or splicing site mutations, one promoter region mutation and one deletion (frameshift). Among them, 10 mutations were previously unreported, including c.734G>T/p.R245M, c.767C>T/p.T256I, c.1039C>T/p.H347Y, c.668C>T/p.P223L, c.1471G>C/p.D491H, c.738-2A>G, c.1045T>C/p.S349P, c.737+8_c.737+9delGCinsTT, c.1070+1G>T, and c.978-1_c.1019del. All missense mutations were predicted as disease-causing by MutationTaster and Polyphen 2. Mutations c.1070+1G>T, c.738-2A>G and c.737+8_c.737+9delGCinsTT are intron codons in close proximity to exons. Mutation c.978-1_c.1019del is frameshift mutation. The most common point mutation was c.698G>A/p.R233H, with a mutation frequency of 9/44 (20.5%) found in five patients with heterozygous mutations and 2 with homozygous mutations. The second most common mutation was c.739G>A/p.G247S, which was found in seven patients from five families with a mutation frequency of 15.9% (7/44). The mutations c.734C>T/p.R245M, c.694C>T/p.Q232X, c.943G>A/p.G315S, and c.457C>T/p.R153X were found in three patients from two families with a genetic frequency of 3/44 (6.8%). Protein TH consists of four subunits. Each subunit is composed of a regulatory ACT domain with an unstructured N-terminal tail of different lengths (11), a catalytic domain and an oligomerization domain (12). In this cohort, 71.1% (32/45, including the heterozygous mutation reported later) of the mutations were located in the catalytic domain (Figure 2). Mutations are detailed in Table 1. Eight patients were siblings from four families, including six sisters and one brother from three families and two brothers from one family. One patient with a homozygous *TH* mutation was a child of consanguineous parents. Six patients were diagnosed with classic DRD and 16 DRD-plus. The average age of onset was 12.1 months. Nine patients were misdiagnosed with cerebral palsy, two with epilepsy, one with muscular dystrophy, and one with metabolic disease. Ten patients had diurnal fluctuations or improvement by sleep or rest. Eight patients had parkinsonism in the course of the disease.

**Table 1:** Clinical manifestations of patients with *TH* mutation.

	**Diagnosed year**	**Gender**	**Family history**	**Age of onset**	**Diurnal fluctuation**	**Site of onset**	**Diagnosis**	**Gene locus**	**Amino acid alteration**	**L-dopa** **mg/kg/d**	**Misdiagnosed**	**Outcome**
1	2005	M	No	7m	No	Generalized	DRD-plus	c.698G>A c.385C>T	p.R233H p.R129X	3.3		Concentration deficiency, easily tired
2	2008	F	Yes	8m	No	Generalized	DRD-plus	c.734G>T c.457C>T	p.R245M p.R153X	3.0	Cerebral palsy	Abnormal posture
3	2008	M	Yes	15m	Yes	LL	Classic DRD	c.734G>T c.457C>T	p.R245M p.R153X	2.5	Cerebral palsy	Normal
4	2011	M	Yes	0m	No	Generalized	DRD-plus	c.739G>A c.1045T>c	p.G247S p.S349P	5.7	Cerebral palsy	Concentration deficiency
5	2011	M	Yes	0m	No	Generalized	DRD-plus	c.739G>A c.1045T>c	p.G247S p.S349P	4.0	Cerebral palsy	Concentration deficiency
6	2012	M	No	12m	Yes	Generalized	DRD-plus	c.698G>A c.698G>A	p.R233H p.R233H	1.3	Cerebral palsy	Concentration deficiency, iPoor coordination
7	2013	M	No	4m	No		DRD-plus	c.698G>A c.1070+1G>T	p.R233H	6.7	Epilepsy	Normal
8	2013	M	No	4m	Yes	Generalized	DRD-plus	c.698G>A c.943G>A	p.R233H p.G315S	4.7	Metabolic disease	Normal
9	2014	F	No	18m	No	LL	Classic DRD	c.767C>T IVS9-1_c.1019del	p.T256I	3.0	Cerebral palsy	Normal
10	2015	M	No	10m	Yes	Generalized	DRD-plus	c.698G>A (G-A)-70 to initiation codon	p.R233H	2.5		Drowsiness, easily tired, poor coordination
11	2015	F	No	0m	No	Generalized	DRD-plus	c.1471G>C c.739G>A	p.D491H p.G247S	3.4		Wide-based gait, Poor coordination
12	2015	F	No	1m	No		DRD-plus	c.1039C>T c.738-2A>G	p.H347Y	1.0	Epilepsy	Sever hypotonia, drooling, Mental retardation
13	2016	M	Yes	48m	Yes	LL	Classic DRD	c.1481C>T c.943G>A	p.T494M p.G315S	3.0		Normal
14	2016	F	Yes	0m	No	Generalized	DRD-plus	c.1481C>T c.943G>A	p.T494M p.G315S	6.5	Cerebral palsy	Abnormal postures
15	2016	M	No	19m	Yes	LL	Classic DRD	c.1240G>A c.1083C>G	p.G414R p.H361Q	5.0	Cerebral palsy	Normal
16	2016	F	No	0m	No	Generalized	DRD-plus	c.739G>A c.694C>T	p.G247S p.Q232X	3.0	Cerebral palsy	Wide-based gait
17	2016	M	No	1m	No		DRD-plus	c.698G>A c.457C>T	p.R233H p.R153X	6.3		Wide-based gait
18	2017	M	No	0m	Yes	Generalized	DRD-plus	c.734G>T c.668C>T	p.R245M p.P223L	4.2		Concentration deficiency
19	2017	F	Yes	0m	Yes	Generalized	DRD-plus	c.739G>A c.694C>T	p.G247S; p.Q232X	2.6		Abnormal posture
20	2017	M	Yes	20m	Yes	LL	Classic DRD	c.739G>A c.694C>T	p.G247S p.Q232X	2.1	Muscular dystrophy	Normal
21	2018	F	No	13m	Yes	Generalized	DRD-plus	c.698G>A c.698G>A	p.R233H p.R233H	1.1		Poor coordination
22	2018	M	No	87m	No	UL	Classic DRD	c.739G>A c.737+8_c.7 37+9delGCi nsTT	p.G247S	2.5		Normal
23	2017	M	No	4.5m	Yes	Generalized	DRD-plus	c.1481C>T heterozygous mutation	p.T494M	4.6		Wide-based gait, drooling

*LL, lower limbs; UL, upper limbs*.

### Patients Diagnosed With Classic DRD With TH Mutation

Six patients were diagnosed with classic DRD. The average onset age was 34.5 months, ranging from 15 to 87 months. They could control their heads at an average age of 3 months, ranging from 2 to 4 months old; turn over at an average age of 4.2 months old, ranging from 4 to 5 months old; sit independently at an average age of 6.8 months, ranging from 6 to 8 months; and walk independently 17 months, ranging from 13 to 25 months. Dystonia, as the main symptom, started from the lower limbs in five patients and from the right arm in one patient. Additionally, four patients had diurnal fluctuations, three had parkinsonism, and one had fever before the onset of dystonia. Three were misdiagnosed with cerebral palsy, and one was misdiagnosed with muscular dystrophy. They had taken an average dosage of 3.0 mg/kg/d (range 2.1–5.0 mg/kg/d) of L-dopa for 47.3 months (range 8–120 months). All of them achieved complete remission without obvious side effects.

Interestingly, three boys with *TH* mutations were diagnosed with classic DRD, and their sisters, who had the same mutations, were diagnosed with DRD-plus.

### Patients Diagnosed With DRD-plus With TH Mutation

Sixteen patients were diagnosed with DRD-plus, including two brothers from one family. The onset age of the patients ranged from neonate to 13 months old, and the average age was 3.8 months old. Six patients were misdiagnosed with cerebral palsy, and two were misdiagnosed with epilepsy. Six patients had diurnal fluctuations or improvement by sleep or rest. Five patients had parkinsonism in the course of the disease.

Among them, seven were born with generalized dystonia and severe developmental delay. Five of the seven patients could control their heads at an average age of 19.4 months, ranging from 4 to 6 years old; turn over at the average age of 13 months, ranging from 7 to 2 years old; and sit independently at the average age of 22.3 months, ranging from 11 to 4 years old. Only one patient (case 16) could walk with support at the age of 20 months old. Two of the seven patients were never able to control their heads; they could roll and sit when they were diagnosed with DRD-plus at the age of 11 years old and 15 months old, respectively. Three of the seven patients had fever-induced encephalopathy with manifestation of lethargy and severe hypotonia, losing all motor function, including head control, turning over, and sitting.

Six patients had relatively normal development before the onset of the disease. All of them could control the head, five could sit independently, 4 could climb or roll over, and two could stand with support. The onset age ranged from 4 to 13 months, with an average age of 9 months. The patients became severely hypotonic and lost almost all the acquired motor function quickly, including head control and sitting. The regression was induced by fever in three patients. Among them, one patient had fever-induced generalized hypotonia and tremors twice. The other three patients did not have any obvious inducing factors.

Three patients had episodic abnormalities, such as oculogyric crises, hypersalivation, tremor, irritability, repeated vomiting, and rigidity, with an early-onset age of 1–4 months. The symptoms were induced by fever in one patient, and there were no obvious causative factors in the other two patients.

All patients had taken L-dopa 1.0 to 6.7 mg/kg/d (average 3.7 mg/kg/d) for 49.9 months (range 9 months to 11 years). Fifteen patients had a dramatic response; however, only two of these patients became completely normal, two patients retained abnormal posture because of delayed treatment, and 11 patients still had symptoms such as concentration deficiency, dysarthria, poor coordination, lethargy, fatigue, and abnormal gait. No improvement was achieved in one patient. The patient (case 12) had poor sucking and repeated vomiting accompanied by episodic head backward, limb stiffness and staring when she was 1 month old. Genetic testing was performed in China and Japan and found the same TH mutations of c.1039C>T/p.347H>Y and c.738-2A>G. The symptoms worsened after L-dopa was added.

### Patient Disease Is Probably Caused by TH Mutation

In one patient (case 23), a heterozygous *TH* gene mutation in c.1481C>T/ p.T494M inherited from his asymptomatic mother was found. The mutation test was repeated several times by different companies using whole-exome sequencing, Sanger sequencing and MLPA testing of *TH* and *GCH1* genes. No mutations, including large fragment deletions, were found in the other allele of the *TH* gene. According to his clinical manifestation, DRD-plus was diagnosed. He could control his head at 3 months old, turn over and had eye contact before the onset of the disease. At 4.5 months of age, generalized hypotonia and tremors presented after fever. He lost the ability to control his head and turn over within several days. A low dose of L-dopa (4.6 mg/kg/d) was used for 19.5 months. The tremors disappeared, and the hypotonia improved dramatically 3 days after the treatment. He could sit independently at 6 months old, walk at 1 year and 5 months old, and verbally communicate at 1 year and 7 months old. However, he still had wide-based gait and drooling at 2 years of age. He had diurnal fluctuation and parkinsonism in the course of the disease.

**Figure 3:**
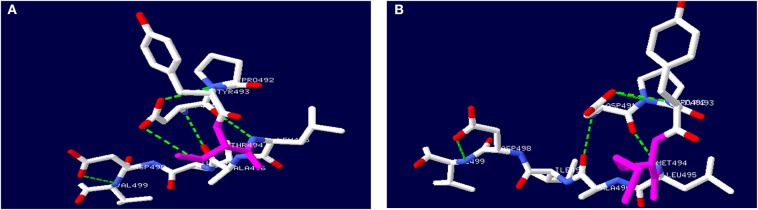
Secondary structure prediction of TH protein by Swiss PDB software. **(A)** showed wild-type TH protein. **(B)** showed mutated TH protein with c.1481C>T, p.T494M gene mutation.

The *TH* gene mutation of c.1481C>T/p.T494M is located in the oligomerization domain. Secondary structure prediction of TH protein with this mutation by Swiss PDB software is shown in Figure 3. The mutation causes the hydrogen bond between aspartic acid (491) and methionine (494) to break. No large deletion was found after MLPA. His parents refused other tests, including CSF, blood or urine, because these tests had no use for treatment.

### Mutations in the GCH1 Gene or PTS Gene

Two patients (6.5%) had *GCH1* gene mutations and were diagnosed with classic DRD, with dystonia beginning in the lower limbs, diurnal fluctuation, improvement by sleep or rest and a sustained response to L-dopa. One patient (a girl, case 24) with a family history of dystonia had dystonia that started from her right toes when she was 2 years old after fever. Her father had the same *GCH1* gene mutation of c.631_632delAT (p.Met211ValfsTer38). He had parkinsonism after extensive writing when he was 27 years old. After taking L-dopa, his symptoms disappeared. Detailed information is shown in Table 2.

**Table 2:** Clinical manifestations of Patients with *GCH1* and *PTS* mutations.

	**Diagnosed year**	**Gender**	**Age of onset**	**Diurnal fluctuation**	**Family history**	**Diagnosis**	**Urine biopterin**	**Urine neopterin**	**Gene**	**Gene locus**	**Amino acid alteration**	**L-dopa mg/kg/d**	**Outcome**
24	2002	F	2y	Yes	Yes	Classic DRD			GCH-1	c.631_632delAT	p.Met211Valfs Ter38	0.4	Normal
25	2016	M	5y	Yes		Classic DRD			GCH-1	c.548A>C	p.E183A	1.8	Normal
26	2014	M	10y11m	Yes			Decreased	Normal	PTS	c.166G>A c.286G>A	p.V56M p.D96N	1.9	Wide-based gait
27	2016	F	3y	Yes			Decreased	Normal	PTS	c.272A>G c.286G>A	p.K91R p.D96N	1.0	Wide-based gait

Two patients had *PTS* gene mutations. The main symptoms included drowsiness, sleeping 15–16 h daily, episodic abnormal behavior and movement such as tremor, stiffness, bradykinesia, drooling, stereotyped movement, involuntary smile, and difficulty talking. Both had diurnal fluctuations or improvement by sleep or rest. The symptoms were induced by fever in one patient. These symptoms presented when they were tired or emotionally stressed at the beginning and became increasingly frequent. They were prone to tiring. All of them were believed to be epileptic. There was no response to antiepileptic drugs such as valproate sodium, topiramate and levetiracetam and no epileptic discharge on EEG during the attacks. They underwent urinary pterin spectrum analysis, and they both had decreased biopterin concentrations and normal neopterin concentrations. After confirming the diagnosis of pyruvoyl-tetrahydropterin synthase deficiency, L-dopa was given at doses of 1 and 1.9 mg/kg/d with no response. The symptoms were significantly improved only after administration of tetrahydrobiopterin (1.88 mg/kg/d). To date, none of the patients had obvious side effects after treatment for 3 and 5 years. Detailed information is shown in Table 2.

### Patients Without Mutation or Without Genetic Testing

In 1 patient (case 28) with classic DRD, no mutation was found by whole-exome sequencing, Sanger sequencing or MLPA of the *TH* and *GCH1* genes. Three patients did not have a gene test. One patient (case 28) had fever before dystonia began. Two patients were misdiagnosed with cerebral palsy, and one was misdiagnosed with epilepsy. Two patients (case 28 and case 31) had typical symptoms of classic DRD, such as dystonia beginning from the lower limbs, mild parkinsonism, diurnal fluctuation and improvement after sleep or rest, and sustained response to low doses of L-dopa. The other two cases (case 29 and case 30) were older brothers and younger sisters. They had similar symptoms, such as early onset at 3 months old, diurnal fluctuation, parkinsonism and episodic staring, head tilting, and gnathospasmus. A low dose of L-dopa was administered after the diagnosis of DRD-plus, and the patient achieved quick remission. Detailed information is shown in Table 3.

**Table 3:** Clinical manifestations of patients without mutation or without test.

	**Diagnosed year**	**Gender**	**Age of onset**	**Site of onset**	**Diurnal fluctuation**	**Family history**	**Diagnosis**	**Gene locus**	**L-dopa mg/kg/d**	**Outcome**
28	2012	M	7y5m	LL	Yes	No	Classic DRD	Negative	8.7	Normal
29	2009	F	3m		Yes	Yes	DRD-plus	No test	3.5	Normal
30	2009	M	3m		Yes	Yes	DRD-plus	No test	2.3	Normal
31	2010	M	1y	LL	Yes	No	Classic DRD	No test	3.5	Normal

*LL, lower limbs*.

## Discussion

**Figure 4:**
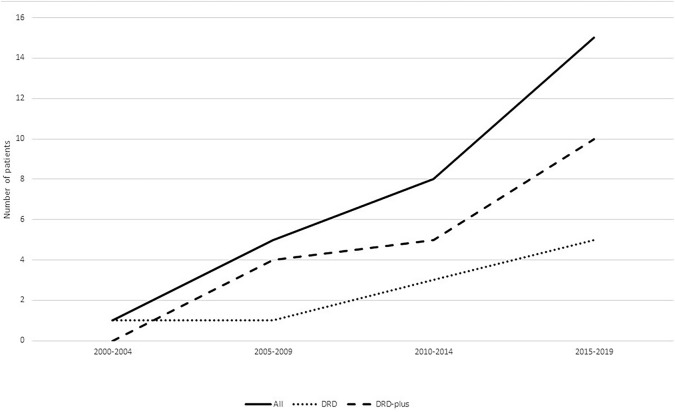
Patients diagnosed with DRD in different years. With the recognition of the disease, more and more patients were diagnosed especially patients diagnosed with DRD-plus.

DRD is a disease with typical manifestations of childhood or adolescent onset, marked diurnal fluctuations and improvement by L-dopa. With the recognition of the disease, various atypical presentations and variable onset ages have been reported. DRD-plus can be diagnosed as patients with dystonia and other atypical symptoms, such as convulsion, ptosis, severe dystonia and autonomic symptoms. Clot et al. (13) reported 64 patients, of whom 57 (89.1%) had classic DRD, and 7 (10.9%) had DRD-plus. The subtype distribution is different from this cohort of 31 patients, in whom 10 patients (32.3%) had classic DRD, 19 (61.3%) had DRD-plus, and two had 6-pyruvoyl-tetrahydropterin synthase deficiency (6.4%). With the development of next-generation sequencing (NGS), more patients with atypical symptoms of DRD had been diagnosed (Figure 4). There was also a difference in gender distribution between this cohort and the reported cohort of Nygaard. In his report, females were affected 2.5–4 times more than males (13, 14). In this cohort, males were 1.9 times more common than females. In classic DRD, the ratio of males to females is 4:1, and in DRD-plus, the ratio of males to females is 11:8. Moreover, the GCH1 mutation was the most common gene mutation; however, in this cohort, the *TH* gene mutation was the most common. The ratio of *TH* to *GCH1* mutation was 11:1. We suppose these differences may be attributed to racial background. Unfortunately, no reports were found to support this supposition.

Genes related to tyrosine hydroxylase, biosynthesis and recycling of BH4, including *GCH1, TH, PTS, SPR, PCBD*, and *QDPR*, were thought to be the causative genes of DRD. In Clot's report, the most common pathogenic gene is *GCH1* (13), with autosomal dominant heredity (2, 13, 15–17). The diseases related to the *GCH1* mutation were classified into three different types (18): (1) autosomal dominant heredity characterized by classical DRD and always accompanied with no hyperphenylalaninemia (HPA) because the autosomal dominant *GCH1* mutation has selective defect expression in the brain and not in the liver (9). (2) Autosomal recessive *GCH1* deficient with HPA. The patients always have severe neurologic disorders, such as psychomotor retardation and convulsions. (3) Compound heterozygous mutation displayed between the above. In Clot's report (13), 47 patients (73.4%, 47/64) carried a heterozygous mutation in *GCH1*. However, in our cohort, there were only two patients (7.1%, 2/28) with heterozygous *GCH1* mutations, in accordance with autosomal dominant inheritance. The manifestations belonged to classic DRD without HPA. Clinical heterogeneity presented in a family (case 24). Males have a later onset age, milder phenotype, and lower penetrance than females, which has also been previously reported (19, 20). Twenty-two patients (78.6%, 22/28) had compound heterozygous mutations or homozygous mutations of the *TH* gene in accordance with autosomal recessive inheritance, which was the most common genetic mutation in this cohort. TH is the limiting enzyme of catecholamine neurotransmitters (21). The phenotypes caused by *TH* gene mutation have been summarized as follows: (1) progressive infantile encephalopathy (22, 23), which refers to patients who mainly showed motor retardation, fluctuating extrapyramidal and autonomic symptoms that treatment with L-dopa could ameliorate symptoms but usually does not normalize (23), (2) classic DRD (24–26), and (3) L-dopa-responsive infantile parkinsonism with good response to L-dopa that was limited by dyskinesia, which refers to patients who had early-onset severe motor problems, including parkinsonism and myoclonic jerks (27). The first and third types were classified as DRD-plus. In the largest published study of 36 patients with TH mutations, the phenotype was classified into two major types (28): type A: progressive hypokinetic-rigid syndrome with dystonia; and type B: complex encephalopathy. The onset age of type A is usually before 1 year of age (range 2 months−5 years old) (13, 22, 26, 29–33), with slowly progressive paroxysmal dystonia and rare additional symptoms (tremor, ptosis, oculogyric crises). The onset age of type B is usually earlier (at birth or within a few weeks of birth) and develops more quickly than type A (23, 34, 35). The onset symptoms are complicated, but they finally develop into marked hypokinesia, bradykinesia and dystonic symptoms. Type B might be accompanied by mental retardation and autonomic functions (36). Type A was similar to classic DRD, and type B was similar to classic DRD-plus in this study.

In this cohort, 72.7% (16/22) of the patients with homozygous or compound heterozygous *TH* gene mutations were DRD-plus. Only 27.3% (6/22) were classic DRD. Fourteen of them were boys, and 8 were girls, with a male to female ratio of 1.75:1. Eight patients had a family history. Six patients were older sisters and younger brothers from three families. However, all the older sisters were DRD-plus, and the younger brothers were classic DRD. This means that the same mutation can result in different manifestations, and females had more severe symptoms than males. The causes of these results need further study. The other two patients were brothers. They had similar manifestations and were diagnosed with DRD-plus.

Only a heterozygous mutation of the *TH* gene was found in a patient with DRD-plus by an extensive gene mutational test, which was inherited from his asymptomatic mother. The mutation c.1481C>T/p.T494M was first reported by Swaans et al. (26). In his report, the mutation was found in two patients with classic DRD from the same family, which were compound heterozygous mutations of c.1010G>A/p.R337H and c.1481C>T/p.T494M. However, only a heterozygous mutation was found in our patient. Whether there is a mutation in the non-coding region of the other allele or there are other mechanisms is unknown. No data about incomplete penetrance have been reported to date. TH belongs to BH4-dependent aromatic amino acid hydroxylases and consists of four identical subunits, with each subunit consisting of three domains (11). TH needs an enzyme-bound non-heme ferrous iron (Fe^2+^), 6R-tetrahydrobiopterin (BH_4_) as a cofactor, and molecular oxygen (O_2_) as an additional substrate for catalysis. Functional characterization and *in silico* analysis showed that the mutation c.1481C>T caused the hydrophilic threonine substituted by a hydrophobic methionine, which led to the breaking of the hydrogen bond and the changing of the α-helix. Finally, tetramer formation was influenced (Figure 3). Whether the severely damaged TH protein structure alone through heterozygous mutation causes the disease needs further study. However, this could not explain why the mother with the same mutation had no symptoms. We presumed that there may be a mutation in the promoter, enhancer, or intron in the other allele.

Generalized dystonia with marked diurnal fluctuation or dystonia presented in the eyelids, mandibular region, trunk, and extremities was observed in patients with *PTS* gene mutations. Other clinical features, such as severe hypotonia, bedridden status, dysphagia, lack of eye contact, small for gestational age, failure to thrive, microcephaly, odd smell, blond hair, frequent pneumonia, hyperthermia, and HPA, were reported (37). PTS mutations are often accompanied by HPA and can be diagnosed early and treated with dopamine and/or BH4, which prevents DRD-like symptoms (13, 38). Only 7.1% of patients (2/28) were found to have compound heterozygous mutation *PTS* mutations in this cohort. They did not have a good response to L-dopa and required BH4 to normalize the symptoms and phenylalanine concentration, as reported in the literature (37). They were included because of *PTS* mutations. Disease caused by *PTS* mutation was recently categorized as dopa-reactive dystonia, but a low dose of L-dopa could not ameliorate patients' symptoms. Dystonia caused by *PTS* mutation requires the treatment of both L-dopa and BH4, which do not meet the clinical diagnosis of DRD. Therefore, whether the disease caused by *PTS* mutation should be classified as dopa-responsive dystonia is debatable. Other gene mutations were not found in this cohort because these enzyme deficiencies result in HPA, which can be detected and treated early to prevent the onset of the disease.

DRD has broad clinical heterogeneity that is often misdiagnosed as diplegic cerebral palsy or hereditary spastic paraplegia due to the brisk lower limb tendon reflexes, increased lower limb tone and spurious striatal plantar response (39). DRD can also be misdiagnosed as epilepsy because of episodic dystonia and abnormal behavior (40). In this study, 11 patients had been misdiagnosed with cerebral palsy, and five patients had been misdiagnosed with epilepsy. Compared with DRD, cerebral palsy is a static disease other than a progressive disease, does not respond to L-dopa administration and has characteristic patterns in neuroimaging that are not observed in DRD. Overall, once dystonia is displayed in patients, we should let patients take a low dose of L-dopa to avoid misdiagnosis. With regard to the differential diagnosis with epilepsy, it is known that interictal and specially ictal EEG are useful to identify epileptic seizures; additionally, ictal and post-ictal epileptic manifestations may have specific features which are not encountered in DRD.

According to the literature, DRD can always be treated with L-dopa, but residual symptoms can be observed. A low dose of L-dopa (1–5 mg/kg/d) treatment had a significant effect, especially on the symptoms of dystonia. L-dopa should be started at 1 mg/kg/day, and it is advisable to add 1 mg/kg over several days or weeks. A higher dose of dopamine, 20 mg/kg/d in children or 1,000 mg/day in adults, could be used in patients diagnosed with classic DRD or DRD-plus (41, 42). The response of L-dopa is usually stable; it is rare for patients to develop dyskinesia, but dance-like movements or athetosis occasionally appear (41), and the rate is not known. Although a dramatic response can be observed in patients with *GCH1* mutations, some still have residual motor symptoms (females are more common), such as dystonia and parkinsonism (43, 44). L-dopa used to treat autosomal recessive GCH1 deficiency requires high doses in childhood (45). Patients with autosomal recessive *GCH1* mutations might need to be treated with BH4 and 5-hydroxytryptophan (the precursor of 5-HT) in addition to L-dopa (46). In patients with *TH* mutations, the response to L-dopa is different, which is contrary to the severity of symptoms (28). For the symptoms of the non-motor system presented in DRD-plus, the response to L-dopa is not as good (31). However, there have been reports of L-dopa-related dyskinesia in patients with *TH* mutations in the early stages of treatment (47, 48). The pathophysiological basis of dyskinesia in DRD is unclear (49). When these situations occur, reducing L-dopa and oral amantadine may be helpful (41, 47). Hwang et al. reported patients with dyskinesia after taking L-dopa 11.6 years (0.5–25 years), with a daily mean L-dopa dose of 343.8 mg/d (range 100–600 mg/d). The amount of L-dopa should be gradually reduced over time if taking the drug over a prolonged period to avoid dyskinesia and recurrent DRD symptoms (41). In this study, the dosage of L-dopa ranged from 0.4 to 8.7 mg/kg/d. Normal posture and movement were achieved in 10 patients diagnosed with classic DRD after a low dose of L-dopa for 8 months to 16 years without obvious side effects. In 19 patients with DRD-plus, L-dopa was used for 9 months to 10 years; four recovered completely, and 14 responded well, though they still had problems such as imbalance, abnormal posture, wide-based gait, concentration deficiency, fatigue, excessive drooling, hypotonia, drowsiness, and mental retardation. Only one patient had no response, and the reason was unknown. Patients in this cohort did not have obvious side effects.

In conclusion, there is broad genetic and clinical heterogeneity in DRD. In recent years, the disease has been diagnosed more easily since NGS has been commonly used, and the atypical manifestations have been recognized more clearly. Several gene mutations could cause DRD. In our study, the most common mutation was in the *TH* gene. Patients with classic DRD have good and sustained responses to low doses of L-dopa. In patients with DRD-plus, although most of them had a good response, some non-motor symptoms remained. Delays in diagnosis and treatment can lead to irreversible abnormalities, so enhancing clinicians' knowledge about DRD is very important.

## Data Availability Statement

The data used and/or analyzed are available from the corresponding author.

## Ethics Statement

This study was approved by the Clinical Research Ethics Committee, Peking University. Written informed consent was obtained from parents of participants or participants of the study.

## Author Contributions

XB designed and conceptualized the study and revised the manuscript. YC designed the study, collected patient information, and drafted the manuscript. YW, JW, QZ, and JY provided help in the genetic analysis and collection of patient information. All authors have approved the publication of the manuscript.

### Conflict of Interest

The authors declare that the research was conducted in the absence of any commercial or financial relationships that could be construed as a potential conflict of interest.
